# Alpha-Synuclein and Cognitive Decline in Parkinson Disease

**DOI:** 10.3390/life11111239

**Published:** 2021-11-16

**Authors:** Tian-Sin Fan, Sam Chi-Hao Liu, Ruey-Meei Wu

**Affiliations:** 1 Department of Neurology, National Taiwan University Hospital Hsin-Chu Branch, Hsinchu City 300, Taiwan; tsfan119@ntu.edu.tw; 2 Department of Neurology, National Taiwan University Hospital, College of Medicine, National Taiwan University, Taipei City 100, Taiwan; samntuh@ntuh.gov.tw

**Keywords:** Parkinson disease, alpha-synuclein, cognition, dementia, non-motor symptom, Lewy body, genetic, *SNCA*, neurodegeneration, biomarker

## Abstract

Parkinson disease (PD) is the second most common neurodegenerative disorder in elderly people. It is characterized by the aggregation of misfolded alpha-synuclein throughout the nervous system. Aside from cardinal motor symptoms, cognitive impairment is one of the most disabling non-motor symptoms that occurs during the progression of the disease. The accumulation and spreading of alpha-synuclein pathology from the brainstem to limbic and neocortical structures is correlated with emerging cognitive decline in PD. This review summarizes the genetic and pathophysiologic relationship between alpha-synuclein and cognitive impairment in PD, together with potential areas of biomarker advancement.

## 1. Introduction

Parkinson disease (PD) is the second most common neurodegenerative disease. Pathologically, it is characterized by dopaminergic neuronal loss in the substantia nigra pars compacta (SNpc), and the proteinaceous aggregates composed predominantly of alpha-synuclein (αS), known as Lewy bodies, within neuronal cell bodies [1]. PD is now recognized as a multisystem disorder with the cardinal motor symptoms bradykinesia, rigidity, resting tremor, postural instability, and various non-motor symptoms (NMS) including anxiety, depression, sleep disorders, autonomic dysfunction, and cognitive impairment [2,3].

Cognitive decline is among the most common and debilitating NMS. There is growing evidence that most patients with PD eventually develop cognitive deficits over the progression of the disease [4,5,6,7]. Mild cognitive impairment in PD (PD-MCI) refers to the transitional state between normal cognition and overt dementia [8]. A study reported the point prevalence of PD-MCI to be around 20–30% [4]. In comparison with age-matched normal control groups, people with PD have an almost six-fold increased risk of developing dementia (PD dementia, PDD), in which they exhibit cognitive decline, especially in executive function, attention, visuospatial domains, and memory [9,10]. The long-term cumulative prevalence of dementia is 75% for PD patients who live for more than 10 years after disease onset [11,12].

The progressive nature of the cognitive impairment may be a reflection of the spreading pathology that underlies PD. The pathophysiological mechanisms of PDD are being elucidated, but likely include the direct cortical involvement of Lewy pathology. Good evidence from postmortem studies shows a correlation between limbic and cortical spread of Lewy pathology in PDD [13,14,15,16,17]. In our daily practice, the progression of motor and non-motor symptoms in PD varies significantly. PD patients who maintain good motor function for years can develop a rapid deterioration in quality of life and reduced life expectancy as cognitive function declines [18,19]. Treatment to halt the progression of cognitive impairment in PD is urgently needed.

In this review, we focus on why PD patients eventually develop cognitive decline and the presumed role of αS, including the contribution of genetics, αS protein–protein interaction, and αS cell-to-cell propagation. Finally, we summarize recent efforts toward the identification of biomarkers for patients with PDD.

## 2. Alpha-Synuclein, Lewy Body, and Dementia

Synucleinopathy underlies a wide spectrum of clinical syndromes, including PD, PDD, dementia with Lewy bodies (DLB), multiple system atrophy (MSA), and pure autonomic failure (PAF). In order to provide diagnostic accuracy and define patients likely to respond to disease modifying therapy, a hierarchical classification has been proposed based on the underlying pathological protein deposition (αS), cellular inclusions (Lewy bodies or glial cytoplasmic inclusion, GCI), and clinical phenotypes (parkinsonism, dementia, or autonomic failure) [20]. The pathological hallmark of MSA is the presence of GCI in oligodendrocytes. In PAF, there is predominantly a peripheral deposition of Lewy bodies in autonomic ganglia and nerve fibers without evidence of central nervous system (CNS) dysfunction other than rapid eye movement sleep behavior disorder (REMSBD). Patients with PAF have an increased risk of developing PD, DLB, or MSA later in life [21], possibly indicating a pathophysiological disease continuum. REMSBD is a well-recognized prodrome of synucleinopathies [22], as well as a risk factor of developing cognitive impairment [23].

The Lewy body is a hallmark pathological feature in familial PD, sporadic PD, and other Lewy body diseases (LBD), including PDD and DLB [24,25,26]. They share αS aggregation and cellular inclusions of Lewy bodies as their key pathogenic events [27,28]. PDD and DLB are together known as Lewy body dementia, and the timing of dementia relative to the clinical features of parkinsonism is the major clinical distinction between PDD and DLB. PDD describes dementia that occurs at least one year after the onset of well-established PD (the one-year rule) [29], whereas in DLB, dementia essentially precedes or co-occurs with parkinsonism and has core features of cognitive fluctuation and visual hallucination [30]. Although PDD and DLB share many overlapping clinical and pathological features, there are major differences. Histopathologically, limbic and neocortical involvement of Lewy pathology are both found in PDD and DLB. However, there is a higher burden of neocortical and limbic LBs, more prominent cortical atrophy, and a higher prevalence of coincident Alzheimer’s disease (AD) pathology in DLB compared with PDD [31,32]. The propensity for LB propagation by seeding may differ between PDD and DLB as well [33]. On the other hand, at PDD’s early stage, it shares a similar αS pathology with PD. Clinically, DLB does not begin with PD or PDD. DLB and PDD also differ in cognitive profiles. Memory and language impairments progress faster in DLB, whereas executive dysfunction progresses more quickly in PDD [34]. Controversy still exists as to whether PDD and DLB should be considered as separate disease entities, or as two ends of the LBD spectrum beginning at the Lewy pathology end with incidental Lewy body disease, through to PD, PDD, and DLB with AD at the amyloid pathology end. There is emerging agreement in clinical trials and research settings that PDD and DLB should be distinguished as two syndromes.

Another emerging concept connecting pathophysiology and cognitive function in neurodegenerative diseases is oscillopathies, which refer to conditions characterized by the abnormal synchronization of synaptic activity [35]. Accumulation of αS can alter synaptic structure and function, in turn impairing the physiological transmission through the cortico–basal ganglia–thalamic circuits, accounting for abnormalities in motor and cognitive function. Mitochondrial dysfunction is one of the putative mechanisms in many neurodegenerative disorders. The generation of neuronal oscillations highly relies on mitochondrial energy provision. Distinct patterns of brain oscillations may correlate with clinical symptoms and network impairment secondary to physiopathological changes [36].

## 3. Physiological Function and Potential Toxicity of Alpha-Synuclein

**Figure 1:**
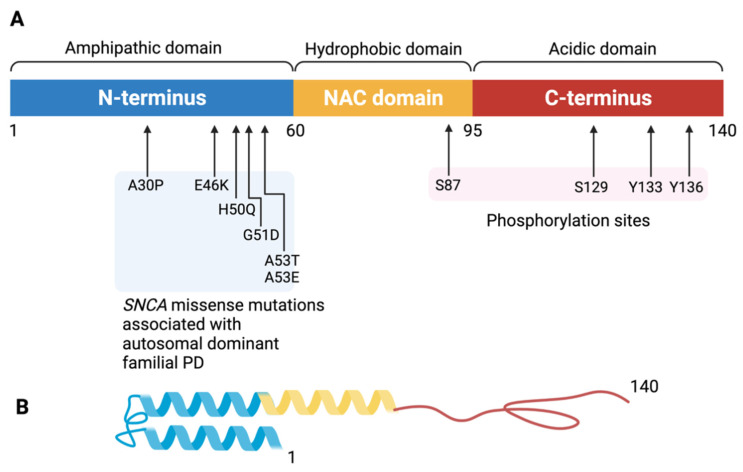
The structure of the alpha-synuclein monomer. (**A**) Schematic depiction of alpha-synuclein structure. The amino acid residues delimiting the N-terminus, NAC region, and C-terminus as well as those that are sites of known mutations are labeled. The 140-amino-acid protein can be divided into three distinct domains. The N-terminal amphipathic domain (in blue) contains the amino acid residues affected by the main alpha-synuclein gene mutations (A30P, E46K, H50Q, G51D, A53T, A53E) associated with autosomal dominant Parkinson disease. The N-terminal region has a helical folding propensity and is responsible for membrane binding. The hydrophobic non-amyloid β-component of plaque (NAC) domain (in yellow) is responsible for promoting aggregation. The C-terminal domain (in red) forms an acidic tail containing the main phosphorylation site at Ser129. The C-terminal domain modulates alpha-synuclein aggregation. (**B**) Tertiary structure of the α-synuclein monomer. Created with BioRender.com (accessed on 12 October 2021).

Alpha-synuclein (αS) is a 140-amino-acid protein localized in presynaptic nerve terminals [37]. It has three domains with distinct biochemical properties corresponding to the amino acid composition (Figure 1). The first 60 residues are known as the N-terminal domain; this region demonstrates α-helical propensity and an amphipathic membrane binding ability [38]. The non-amyloid β-component of plaque (NAC) domain comprises residues 61–95; this region is highly amyloidogenic and responsible for protofibril and fibril formation and aggregation [39]. The carboxyl-terminal (C-terminal) domain, composed of residues 96–140, is the segment where major phosphorylation and truncation occurs. The C-terminal domain of αS limits pathologic misfolding and aggregation due to its structural factors. The negatively charged C-terminal domain works as a self-chaperone to prevent αS fibrillation by interaction with the NAC region [40]. Loss of acidic C-terminal residues through truncation promotes fibril formation [41]. The complete physiological function of αS remains unknown, though it is well established that αS is involved in various neurophysiological processes, including synaptic vesicle recycling, neurotransmission, and synaptic plasticity [42,43,44,45].

αS is normally a soluble protein, but it can aggregate to form insoluble fibrils which, in association with other molecules such as ubiquitin, neurofilament protein, alpha B crystallin, organelles, and lipid membranes, form Lewy bodies [46,47]. αS can exist in the neuron in a monomeric, oligomeric, and soluble protofibrillary state [48]. Monomeric αS is highly dynamic and can populate a large number of different conformational or assembly states [49,50]. In synucleinopathies, the formation of the distinct αS species is determined by the nature of the self-assembly processes, which is influenced by many factors including the alpha synuclein gene (HGNC approved symbol *SNCA*) mutation or multiplication, epigenetic regulation, post-translational modification, micro-environments, etc. [51,52]. The distinct forms of the αS protein stack aggregates in neurons, nerve fibers, or glial cells at different rates, and can lead to mixed fibrillar polymorphs (species) with different intermolecular interactions, surface characteristics, and pathological consequences [53,54,55]. However, the precise connection between αS cluster structure and toxicity remains a subject of intense and controversial discussion [53]. We will summarize the literature in the following paragraphs.

Both the oligomeric and fibrillar forms of αS are toxic to cells, but whether αS oligomers or fibrils are more toxic remains a subject of debate [53]. Growing experimental evidence suggests that specific oligomeric species are the most cytotoxic forms of αS and play a key role in disease [56,57,58]. On the other hand, αS fibrils have also been reported to be toxic and their toxicity has been associated with membrane perturbation [59,60,61]. While oligomers are possibly implicated in the collapse of neuronal homeostasis, the fibrillar state(s) appears to be the most efficient at propagating itself both in vitro and in vivo. While αS oligomers possess toxic properties and are more robust than fibrils, there is no convincing evidence that they can spread in vivo rather than be formed as a collateral effect of the overall aggregation process [62]. In fact, there is no evidence that non-fibrillar oligomers can propagate in a manner similar to that of fibrils [53]. The αS fibrils can continue to aggregate in association with other proteins such as ubiquitin, neurofilament protein, and alpha B crystallin and form Lewy body-like inclusions [63,64]. The mechanistic relationship between oligomers and fibrils remains to be clarified, both in terms of oligomer assembly into fibrils and the potential dissolution of fibrils into oligomers [53].

αS clusters (oligomers and fibrils) can harm cells through various mechanisms, presumably by interacting with other biomolecules and organelles [65]. For example, it has been proposed that αS could interact with synaptic vesicles and synaptic proteins such as phospholipase D2 [66], various members of the family of RAB small GTPases [67], and SNARE complexes [54,68]. αS neurotoxicants can be classified as various channel inhibitors, receptor inhibitors, receptor agonists, synaptic vesicle inhibitors, and many more [51]. The toxicity of αS fibrils and oligomers is in part the consequence of changing the characteristics of lipid membranes. They affect, for example, membrane permeabilization and the formation of pore-like structures [69,70,71,72], lipid diffusion and packaging [73], synaptic vesicle fusion pore size [74], and membrane curvature [75]. The possible targets of αS include synaptic vesicles [76], endoplasmic reticulum (ER)–Golgi transport [77,78], mitochondria [79,80,81], and lysosomes and other proteolytic machinery [82,83,84]. The general principle is that multiple systems can be affected by αS clusters and, if they have a common attribute, they are likely to be lipid membranes [85].

The detrimental effects of αS continue to grow as αS fibrils start to form LBs. The exact mechanisms that promote the aggregation of αS into LBs and what role aggregation plays in pathogenesis remain to be clarified. A time-dependent shift in the morphology and localization of αS pathology from fibrils to cell body inclusions has been demonstrated. The initial aggregation of αS likely starts in presynaptic terminals and accumulates in axons. After reaching the neuronal cell body, αS aggregates recruit more αS monomers, undergo posttranslational modifications, and interact with other cellular components to form mature LBs. LB formation and maturation can cause mitochondrial disassembly, mitophagy, mitochondrial depolarization, and synaptic dysfunction that result in progressive neurodegeneration [63]. These findings also support the well-established concept that mitochondrial accumulation of αS is associated with impaired complex-I-dependent respiration, decreased mitochondrial membrane potential, and increased levels of reactive oxygen species [86,87].

Recent evidence supports a prion-like mechanism of αS aggregation and spread, whereby introduction of exogenous αS pre-formed fibrils causes endogenous αS to progressively adopt an insoluble, aggregated conformation [88,89]. PD patient-derived αS aggregates can also be taken up by neurons and astrocytes and induce different endogenous responses in the two cell types, leading to neuronal death [90]. However, the exact mechanism of the spreading of αS fibrils remains a subject of intense discussion. Some possible pathways may include trans-synaptic transmission, direct membrane penetration, exocytosis and endocytosis, extracellular vesicles (EVs), and tunneling nanotubes [91,92,93,94,95,96].

To sum up, the neurotoxicity of αS aggregates and LB formation can lead to (1) the disintegration of synapses [97,98,99,100], (2) mitochondrial dysfunction, (3) membrane perturbation and dysfunction [101,102,103], (4) αS-induced neuroinflammation via microglial and astrocyte activation [104], and (5) prion-like propagation between neurons. It is also worth noting that the effects of the soluble (normal) form of αS have largely been overlooked, and thus it remains unclear whether the toxicity arises from the accumulation of abnormal αS or the depletion of the soluble (normal) αS.

## 4. Parkinson Disease and Cognitive Decline: Genetic Contribution, Alpha-Synuclein Propagation, and Protein–Protein Interaction

**Figure 2:**
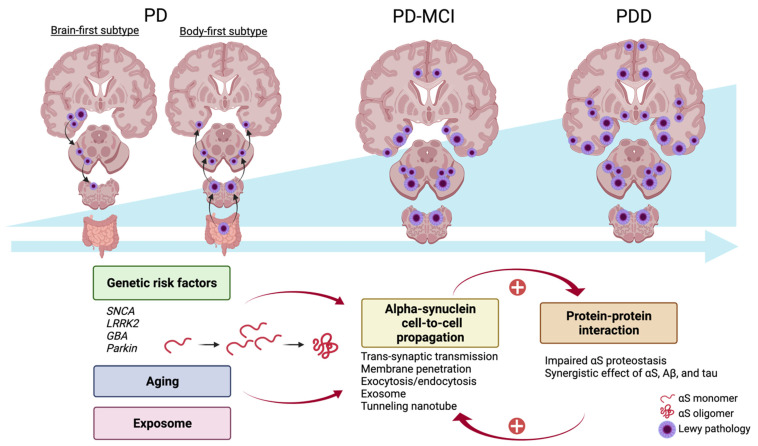
Alpha-synuclein propagation and Parkinson disease dementia. In the early stage of Parkinson disease, the initial alpha-synuclein (αS) pathology originates in the enteric nervous system and spreads to the CNS via the bilateral vagal innervation (body-first subtype) or originates in the unilateral amygdala and spreads to predominantly the ipsilateral hemisphere (brain-first subtype). When the global burden of αS pathology increases with time, indicated by the blue arrow to the right side and the right triangle under the brain figures, patients with Parkinson disease may develop dementia. Genetic mutation, aging, and the exposome—an integrated function of exposure—contribute to an increased burden of αS and consequent fibrillation. αS can spread from cell to cell and interact with both biological proteins and pathogenic proteins such as Aβ and tau. The vicious cycle between αS propagation and dysregulation of protein–protein interaction further enhances neurodegeneration. Aβ, Amyloid-β; αS, alpha-synuclein; CNS, central nervous system. Created with BioRender.com (accessed on 12 October 2021).

As mentioned above, αS aggregation in certain brain regions correlates with cognitive decline in patients with PD. At PDD’s early stage, it shares a similar αS pathology with PD. However, the risk of developing into progressive cognitive decline increases over time as the global burden of αS pathology increases. The progression of PDD is associated with the αS propagation. The relationship between αS burden and PDD is summarized in Figure 2. In this section, we will discuss the factors contributing to cognitive impairment in PDD. These include genetic causes, αS propagation, and protein–protein interaction.

### 4.1. Genetic Contribution

#### 4.1.1. PD-Related Genes

- *SNCA*

The vast majority of PD cases are sporadic, but multiplications of and missense mutations in *SNCA*, the gene encoding αS, have been associated with familial forms of PD. Mutations in the *SNCA* gene impose significant risks of developing dementia. *SNCA* point mutations (A53T, E46K, H50Q, G51D, A53E, and A53V) and *SNCA* triplication are associated with earlier disease onset with faster disease progression and dementia [105,106]. Cortical Lewy pathology is very common with all *SNCA* mutations. In patients with the *SNCA* E46K mutation (E46K-SNCA), there is aggressive and widespread LB deposition across the peripheral nervous system (PNS) and CNS, with early non-motor features, small fiber denervation, severe parkinsonism, and dementia [107,108]. Postmortem brain examinations in patients with the *SNCA* G51D mutation (G51D-SNCA) show significant neuronal loss in the frontal and temporal cortices, hippocampal CA2/3 subregions, SNpc, and DMV, and widespread neuronal αS immunoreactive inclusions, suggesting that αS may contribute to cognitive decline [109]. In a comprehensive MDSGene review, cognitive decline was described in 70% of *SNCA* mutation carriers [110]. A meta-analysis conducted by Marsili et al. evaluated the different timelines to postural instability and other milestones signaling advanced disease, including autonomic dysfunction and cognitive impairment, in monogenic PD. In PD patients with the *SNCA* mutation, the most prevalent milestones is autonomic dysfunction (29%) [111]. Since autonomic dysfunction is a prevalent symptom in PDD [112], it is proposed that the connection between autonomic dysfunction and cognitive impairment is the extensive αS pathology in both PNS and CNS. Apart from *SNCA*, other PD-related genes such as leucine-rich repeat kinase 2 (*LRRK2*), glucocerebrosidase (*GBA*), and *parkin* also have been linked to alterations in αS levels.

- *LRRK2*

Although an appreciable subset of *LRRK2* PD cases display neuronal loss in the SNpc without LB pathology [113], there is emerging evidence for interplay between *LRRK2* and αS. In a postmortem study in PD patients with the *LRRK2* G2019S mutation, cognitive decline was correlated with the presence of LB pathology [114].

Aging affects fundamental cellular machinery by increased oxidative stress and impaired proteostasis [115,116] and is considered a major risk factor for PD. *LRRK2* mutations facilitate PD through several possible mechanisms. Among them, studies have pointed out the role of *LRRK2* in autophagy. Tong et al. demonstrated renal atrophy and accumulation of phosphorylated αS at S129 and ubiquitinated proteins in the kidneys in *LRRK2*^−/−^ mice at 20 months of age [117]. Moreover, Tong and colleagues found an age-dependent biphasic alteration of the autophagic activity in *LRRK2*-/- kidneys, which would in turn result in protein accumulation and aggregation during aging [118]. Since *LRRK2* is a serine-threonine kinase, it has been suggested that G2019S mutant *LRRK2* has increased kinase activity and can phosphorylate αS, resulting in αS aggregation [119]. Recent studies suggest that *LRRK2* is not the main kinase responsible for most αS phosphorylation. Several kinases have been shown to be implicated in phosphorylating αS, such as G protein-coupled receptor kinases and polo-like kinases [120,121]. However, *LRRK2* kinase still plays an important role in modulating αS aggregation in the CNS. Evidence suggests that Rab GTPases are cellular physiological substrates of *LRRK2* kinase. Steger and colleagues found that *LRRK2* phosphorylates endogenous Rab3A/B/C/D, Rab8A/B, Rab10, Rab12, Rab35, and Rab43 in cells through systemic assays using phosphoproteomics [122]. In primary cortical neurons, studies have shown that the phosphorylation of Rab35 mediates mutant *LRRK2*-induced toxicity [123]. In addition, *LRRK2*-mediated Rab35 phosphorylation positively regulates αS breeding, linking *LRRK2* kinase activity to αS aggregation [124]. Moreover, pathogenic *LRRK2* has been shown to alter vesicular trafficking events and endo-lysosomal functioning and to mediate αS propagation by phosphorylating Rab35 [124,125].

- *GBA*

One of the most common lysosomal storage disorders is Gaucher disease (GD), which is caused by a recessively inherited deficiency in β-glucocerebrosidase (Gcase) and subsequent accumulation of toxic lipid substrates. *GBA* mutations are the strongest genetic risk factor for PD and can also increase the risks of other α-synucleinopathies [126,127,128,129,130]. Mutations and variations in the *GBA* gene are associated with more rapid disease progression and a three-fold increased risk for dementia compared with patients with PD [131]. In a pathological examination, widespread neocortical and limbic αS pathology tended to occur more frequently in the PD patients with *GBA* mutations compared with the patients with non-*GBA* mutations [132]. It has been shown that the major Gcase substrate, glucosylceramide, can directly influence the amyloid formation of αS by stabilizing soluble oligomeric intermediates. Functional loss of Gcase activity causes αS accumulation and neurotoxicity through aggregation-dependent mechanisms in primary cultures and human iPS neurons [133]. It is thought that an escalating feedback loop might exist between Gcase and αS that can lead to the self-propagation of disease and eventually dementia. Accumulation of αS can cause inhibition of Gcase by interfering with endoplasmic reticulum-to-Golgi trafficking, which in turn, leads to decreased Gcase activity and promotes the accumulation of αS [133].

- *Parkin/PINK1*

These genes are discussed together as their protein products are linked to mitochondrial function, and their biallelic loss-of-function mutations all cause autosomal recessive young-onset PD [134,135,136]. *Parkin* is an E3 ubiquitin ligase that mediates numerous cellular processes connected to mitochondria. *Parkin* over-expressing animal models are protected against αS toxicity [137,138], suggesting a link between αS and *parkin*. In a transgenic animal model, *parkin* co-expression with αS reduced the levels of phosphorylated αS and attenuated cell death and inflammation [139]. *PINK1* is a serine/threonine kinase acting as a molecular sensor of mitochondrial quality that accumulates on the outer membrane surface of dysfunctional mitochondria where it simultaneously recruits and activates *parkin’s* E3 ubiquitin ligase activity. This triggers multiple signaling events to degrade damaged mitochondria via the mitophagy pathway [120]. *Parkin*-associated PD has been considered a non-LB disorder, with a few reported exceptions in compound heterozygous mutation carriers [140,141,142]. Clinically, patients with a *parkin* or *PINK1* mutation have slower motor and neurocognitive progression [143,144].

#### 4.1.2. Non-PD Related Genes

It has been proposed that other traditionally non–PD-related genetic variations are also accountable for developing PDD. Common genetic variation of the apolipoprotein E (*APOE*) ε4 allele and microtubule-associated protein tau (*MAPT*) H1-haplotype have been linked to earlier development of dementia in patients with PD [145].

- *APOE*

Apart from being the strongest genetic risk factor for late-onset AD, the *APOE* ε4 allele has also been found to be a genetic risk factor for both LBD and PDD [146,147,148,149]. In a postmortem study, *APOE* ε4 has been associated with the severity of LB pathology independently of AD pathology [150]. In a recent case-control GWAS in three large longitudinal PD cohorts, *APOE* ε4/ε4 individuals showed significant cognitive decline over time [151]. In a double transgenic mouse model on an *APOE* knockout (A53T/EKO) or human *APOE* knockin (A53T/E2, E3, or E4), A53T/E4 mice accumulated higher amounts of brainstem αS compared with A53T/EKO and A53T/E3 mice [152]. In a human *APOE*-targeted replacement murine model expressing *APOE2*, *APOE3*, or *APOE4*, delivery of overexpressed human wild-type αS using adeno-associated virus demonstrated that *APOE4* exacerbated αS pathology in the absence of amyloids [153]. To sum up, *APOE* directly regulates αS pathology and hence disease progression towards PDD.

- *MAPT*

Human tau is a microtubule-binding protein encoded by the *MAPT* gene. In different GWAS studies investigating the influence of common genetic variations in PD, *MAPT* is one of the significant associations that have been replicated. The genetic architecture of *MAPT* has been evaluated in a Caucasian European ancestry cohort of PD patients without dementia. There is a statistically significant association between the H1 haplotype and PD risk, especially in non-tremor dominant PD [154]. It is worth noting that co-aggregation of αS and tau filamentous inclusions has been found in human and PD transgenic mice brains and oligodendrocytes. The co-incubation of tau and αS synergistically promotes the fibrillization of both proteins [155]. This suggests that tau may have potential roles in contributing to the development of PDD. *SNCA* and *MAPT* genes interact in affecting risk for PD. Many other studies have discovered that *MAPT* is an independent risk factor for the development of cognitive impairment or dementia in PD patients [156,157]. One study analyzing an *SNCA* risk allele combined with the *MAPT* H1-haplotype suggests they cause a synergistic increase in the susceptibility of developing dementia in patients with PD [158]. Conversely, some studies have reported no genetic interaction between *SNCA* and the *MAPT* polymorphism [159,160,161,162].

#### 4.1.3. Polygenic Risk Score (PRS) in PDD

Polygenic risk score is a numerical indicator that summarizes the estimated risk based on multiple genetic markers associated with a disease or a trait [163]. Both the recent PD GWAS in European [146] and Asian [164] populations have been deployed as references used to build a PD-related PRS. A PD GWAS browser tool (https://pdgenetics.shinyapps.io/GWASBrowser/, accessed on 12 October 2021) has also been created to assist PD research. Several genetic loci have been implicated in influencing cognitive function in PD. In a population-based study, a higher polygenic risk score was associated with faster cognitive decline [165]. This finding suggests that PDD may be induced by the cumulative dysfunction of multiple cellular pathways.

### 4.2. Alpha-Synuclein Propagation

A detailed neuropathological examination by Braak and colleagues demonstrated a sequential caudal-to-rostral progression of αS pathology from the dorsal motor nucleus of the vagus (DMV) in the caudal medulla in the early stage of PD to limbic and neocortical areas in the later stage [166,167]. Emerging evidence also suggests that αS pathology can begin in the enteric or peripheral autonomic nervous system and invade the CNS via retrograde vagal transport (the dual-hit hypothesis). Despite some exceptions, the spatiotemporal progression of αS pathology in the majority of PD patients suggests that αS undergoes prion-like, cell-to-cell transmission within individuals. These hypotheses suppose that both neuronal vulnerability and connectivity are necessary to facilitate the propagation of αS pathology along susceptible neurons.

The olfactory bulb is affected by αS pathology in almost all cases of LBD and is considered as an entry site for prion-like propagation [168]. Since olfactory limbic structures are key components of cognitive processing, αS involvement of olfactory, limbic, and neocortical structures in PD and DLB plays a crucial role in the development of dementia. There is close anatomical proximity of the olfactory bulb with limbic regions and projections to the neocortex. There is a putative olfactory/limbic pathway suggesting that αS can propagate along the neuronal connections and induce neurocognitive impairment as αS pathology reaches the neocortex [169].

In a new hypothesis, PD is divided into *brain-first* (top-down) and *body-first* (bottom-up) subtypes [170]. In the brain-first subtype, an αS seed first develops in the CNS and injures dopaminergic neurons of the SNpc, which are the most vulnerable to αS pathology [171,172]. In the body-first subtype, αS pathology is initially found in the enteric nervous system and ascends via both the left and right DMV, owing to the overlapping parasympathetic innervation of the gut [173]. Thus, the clinical symptoms of the body-first PD subtype are relatively symmetric and exhibit more autonomic prodromal symptoms before the onset of parkinsonism. A recently proposed PD etiopathogenesis model called the Synuclein Origin and Connectome model (SOC model) posits that *α*S pathobiology is the core feature of PD pathogenesis [174]. Moreover, the anatomical origin of the initial synuclein inclusion and dominant ipsilateral connectivity of the human brain may explain motor asymmetry, the presence of non-motor symptoms (for example, constipation and REM stage behavior disorder), and the variable rates of cognitive decline seen in different subtypes of PD. The presence of αS-containing LBs in neocortical and paralimbic regions is one of the main pathological hallmarks correlating with PDD [175,176]. It has been hypothesized that in body-first PD, as the αS pathology propagates symmetrically from the PNS to the CNS, the total burden of αS is higher, so the patients are more likely to develop dementia.

### 4.3. Protein–Protein Interaction

During protein biogenesis, proteins form multiple interactions with other proteins. Dysregulation of biological protein–protein interactions may result in αS aggregation and eventually disease progression. Aggregation could be initiated by the loss of assistance during *α*S biogenesis induced by mutations in *α*S, or a loss of interacting proteins. In a study using Ingenuity Pathway Analysis (IPA, Qiagen), αS-interacting partners can be categorized into seven different functional clusters including transcription, translation, folding/trafficking, modification, secretion, mitochondrial-associated, and degradation [177]. For example, chaperones assist protein folding and inhibit fiber assembly. Direct expression of the chaperone Hsp70 protected against αS toxicity by preventing dopaminergic neuronal loss in Drosophila [178]. Another experiment showed that the chaperonin containing TCP-1 (CCT) can inhibit aggregation by interacting with the NAC domain of αS A53T oligomers [179]. Co-translational and post-translational modifications can affect protein conformation, localization, and degradation. Post-translational modifications of αS include nitration on tyrosine residues [180], ubiquitination [181], phosphorylation [182], C-terminal truncation [183], and SUMO modification [184]. Among them, the most attention has been devoted to phosphorylation at S129. It is still unclear whether αS phosphorylation is a cause or a consequence of aggregation, or whether phosphorylation is neurotoxic or neuroprotective. Whereas only a small fraction (4%) of soluble αS monomer is phosphorylated under physiological conditions in vivo, approximately 90% of deposited αS is phosphorylated at S129 within LBs [182,185,186], suggesting that phosphorylation plays a crucial role in the regulation of αS aggregation, LB formation, and neuronal degeneration. A study using peptide pulldown assays and mass spectrometry revealed differences in the protein–protein interactions of phosphorylated versus non-phosphorylated αS [187]. Phosphorylated peptide containing the pS129 of αS had a higher binding affinity for cytoskeletal proteins, vesicular trafficking proteins involved in endocytosis, and enzymes involved in protein serine phosphorylation. This result suggests that phosphorylation could promote the binding of αS to the synaptic cytoskeleton and regulate the synaptic vesicle cycle. This is compatible with the fact that cortical LBs contain cytoskeletal proteins, including tau [188]. The non-phosphorylated peptide interacted preferentially with mitochondrial complexes I, III, and IV. By replacing S129 with either an alanine (S129A) to block phosphorylation, or with an aspartate (S129D) to mimic phosphorylation, studies have shown that overexpression of S129D αS results in less neuronal death than overexpression of S129A αS [189,190]. These findings suggest that when levels of non-phosphorylated αS are high, the protein may enter mitochondria and induce mitochondrial dysfunction and subsequent cell death.

The clearance of αS involves a variety of extracellular and intracellular degradation systems that require well-regulated enzymatic reactions. Both monomeric and aggregated forms of αS in the extracellular space can be cleared by internalization by neighboring neuronal cells including neurons, astrocytes, and microglia, and be degraded within the lysosome [191,192]. Intracellular αS species are degraded by the complementary works of the proteasomal and lysosomal pathways. In the ubiquitin-dependent proteasome pathway, the enzymes E1, E2, and E3 activate ubiquitin and conjugate a ubiquitin chain to αS. After ubiquitination, the αS-polyubiquitin complex can be recognized by the 26S proteasome and undergo degradation [193]. Ubiquitin-independent proteolysis may also occur for natively unfolded proteins [194]. Using rat pheochromocytoma PC12 cell lines, cells transfected with A53T human protein showed significantly strong staining for ubiquitin in the cytoplasm instead of the low-level diffuse staining seen in the empty vector-transfected or wild-type αS-transfected cell lines. Moreover, the A53T human αS-transfected PC12 cell line showed statistically significant lower proteasomal chymotrypsin-like activity then a wild-type αS-transfected cell line, suggesting the critical role of ubiquitination in proteostasis. The lysosome degrades intracellular proteins through the endosomal and three autophagic pathways: chaperone-mediated autophagy (CMA), macroautophagy, and microautophagy. In CMA, cytosolic chaperone heat-shock cognate 70 (hsc70) selectively recognizes a pentapeptide motif on the target protein (in αS, _95_VKKDQ_99_) and this complex translocates to the lysosomal membrane and binds to the lysosome-associated transmembrane protein LAMP2A for subsequent degradation within the lysosomal lumen. In PC12 cells expressing a mutant αS that lacks the CMA recognition motif and in which Lamp2a was downregulated by use of a small interfering RNA, higher levels of the mutant wild type αS were observed by immunoblotting [195].

Neurodegenerative diseases can have coexistence of different proteinopathies and heterogeneous clinical symptoms. Despite the central role of αS pathology in PDD, coexistent amyloid-β (Aβ) and tau pathologies are also commonly found in neurodegenerative disorders, suggesting possible crosstalk between them. It has been shown that Aβ, tau, and αS fibrils promote the fibrillation and accumulation of one another in vitro [155,196,197]. In a transgenic mouse model expressing human αS, Aβ peptides, or both, double transgenic mice had severe deficits in learning and exhibited motor deficits before αS single-transgenic mice [198]. Another bigenic mouse model with combined pathologies of tau and αS demonstrated exacerbated behavioral impairment associated with accelerated pathological αS and tau [199]. Aβ and tau pathologies independently contribute to the development of cognitive decline in PD but may also act synergistically with αS pathology to confer a worse prognosis [200,201]. Nuclear imaging evidence obtained by using [^18^F]AV-1451 tau PET and [^11^C]PiB amyloid PET demonstrated that cortical tau aggregation was greater in DLB or PDD patients in comparison with PD subjects without cognitive decline and normal controls [202]. To clarify how αS modulates tau spreading in mouse brains, in a recent study on mouse preformed fibrils, αS, enriched AD brain-derived tau, or the two in combination were injected into wild-type mice. αS staining was primarily found in the hippocampus and entorhinal cortex and revealed no major differences in the burden and spreading of αS between groups. On the other hand, tau pathology burden and spreading were significantly accelerated in the presence of αS compared with injected tau alone. These findings point to the important role of αS as a modulator of tau pathology burden [203].

## 5. Alpha-Synuclein as a Biomarker of PDD

### 5.1. αS, Amyloid-β (Aβ), and Tau Pathology in PDD

Some key unmet medical needs in PD include the inability to make a definitive diagnosis at the early stages, the lack of clear disease progression patterns, and the lack of disease-modifying therapies, which are not yet in sight. The natural course of motor and cognitive decline in PD can vary considerably, likely depending on variation in the underlying pathological characteristics. Identifying reliable biomarkers of cognitive decline in PD provides clues to disease progression.

The association between αS levels in various tissues in the body and PD is well established. Cerebrospinal fluid (CSF) is often used as a source of biomarkers in neurological disorders because it may directly reflect changes and disease pathologies in the CNS. Lower αS levels in the CSF have been observed in patients with PD and related synucleinopathies compared with controls in many studies [204,205,206,207,208]. In the DATATOP cohort, lower CSF levels of total αS predicted better preservation of cognitive function in PD patients [209]. Another longitudinal follow-up study observed that higher total αS in CSF was associated with the rapid progression of both motor symptoms and cognitive decline in PD over 2 years [210].

CSF biomarker tests are invasive, making them unsuitable for office-based practice and repeated collection during the course of disease. The diagnostic value of other accessible, non-invasive, and highly reliable biomarker sources requires further investigation. Many studies have shown that plasma or serum αS level correlates with motor severity and cognitive decline in patients with PD [211,212,213]. Although the level of saliva and urine αS did not yield a clear differential value, tear fluid revealed small but significant differences in total αS levels between PD and control subjects [214]. In a study investigating skin biopsies, a significant increase in αS seeding activity was observed in individuals with PD and synucleinopathies compared with controls with tauopathies and non-neurodegenerative diseases [215]. An in vivo study using 3 mm punch biopsies taken from the cervical C7 paravertebral area, thigh, and distal leg showed different skin αS deposition patterns between synucleinopathies. For example, there is higher positivity of phosphorylated αS at S129 in cervical skin samples in PD and DLB. The occurrence of phosphorylated αS staining along skin nerves per sample was higher in DLB than PD. MSA displayed a unique pattern of αS pathology limited in somatosensory skin fibers [216].

As briefly discussed in the previous section, despite the central role of αS pathology in PDD, coexistent amyloid-β (Aβ) and tau pathologies are also commonly found in neurodegenerative disorders. In fact, up to 50% of patients with PDD may exhibit comorbid AD in postmortem studies [217,218,219]. Numerous studies have found that the levels of Aβ plaques and tau tangles are also correlated with cognitive performance in patients with PDD [31,201,220,221]. Yu et al. used ELISA to show that an elevated level of CSF tau is significantly correlated with cognitive impairment in PD patients [222]. Liu et al. followed up the cognitive decline of PD patients for 4.3 years after the initiation of levodopa and found that CSF phosphorylated tau (p-tau) and the p-tau:Aβ42 ratio predict subsequent decline on cognitive tasks involving both memory and executive functions [223]. These results suggest that αS, tau, and Aβ could be used as potential biomarkers for PD patients with cognitive impairment.

### 5.2. MicroRNAs as Biomarkers for PD/PDD

Recently, microRNAs (miRNAs), small non-coding RNAs approximately 21–25 nucleotides in length, have been widely studied for their roles in silencing gene expression and as potential biomarkers for various diseases. Few miRNAs are currently used as biomarkers for PDD. Han et al. recruited 39 PD patients with normal cognition, 37 PD patients with mild cognitive impairment, and 22 PD patients with dementia (PDD), and used RT-qPCR to show that the levels of three miR-29s (miR-29a, miR-29b, and miR-29c) in the PDD group were significantly lower than those in the PD with normal cognition group. A significant association was found between miR-29s and PDD [224]. Yang et al. recruited 30 PD patients with dementia and 208 healthy controls and found a significant difference in miR-135a expression levels between the PDD group and healthy controls [225].

Because the number of PDD biomarker miRNAs is currently small, more research is required to discover new ones. Here we will first list miRNAs that have been reported to be associated with PD and then predict candidate miRNAs for PDD. Recent studies have identified several miRNAs that can directly or indirectly regulate the expression of αS. In PD patients, miR-7, miR-153, miR-34b/miR-34c, and miR214 have been reported to directly bind to *SNCA* transcripts and regulate their modification [226]. Other miRNAs that indirectly impact αS without binding to αS include miR-21, miR-224, miR-373, miR-379, miR-128, let-7, miR-133b, miR-26b, miR-106a, miR-301b, miR-443, miR-15b-5p [226]; miR-124 [227,228,229], miR-let-7d, miR-22, miR-23a, miR-24, miR-142-3p, miR-222 [230]; miR-27a-3p, miR-125a-5p, miR-151a-3p, miR-423-5p, let-7f-5p [231]; miR-1, miR-19b-3p, miR-153, miR-409-3p, miR-10a-5p [232]; and miR-16-1, miR-138-2-3p, miR-205, miR-224, miR-320a, miR-373, miR-379, miR-494, miR-4639-5p [233].

As previously discussed, AD neuropathology (especially tau and Aβ aggregates) appears to play an important role in the pathogenesis of PDD in a significant proportion of patients. The pathomorphologic characteristics of PDD/DLB seem to cover the neuropathological features of both PD and AD [220]. In order to identify more potential biomarkers for PDD, three miRNAs, miR124, miR-7f-5p, and miR153, that are reported to be correlated with both PD and AD, will be briefly introduced. miRNA-124 is downregulated in AD, PD, and HD [227]. A recent study pointed out the potential importance of miR-124-dependent gene networks in PD, as nearly 25% of validated miR-124 targets are deregulated in this disease [234]. miR-124 and miR-7f-5p are also deregulated in the hippocampus of patients with late-onset Alzheimer’s disease [235]. miRNA-153 can directly target *SNCA* transcripts and downregulate αS expression [236]. On the other hand, Liang et al. used a miR-153 transgenic mouse model and showed that miR-153 downregulated the expression of amyloid precursor protein (APP) and amyloid precursor-like protein 2 (APLP2) proteins in vivo [237]. Because of their dual functional roles in both PD and AD, these miRNAs are predicted to be more likely to contribute to PDD pathology.

### 5.3. EV Proteins as Biomarkers for PD/PDD

The αS in the plasma, CSF, and tissue can be derived from extracellular vesicles (EVs) or released from cells via an unconventional protein secretion mechanism [96,238]. However, αS derived from the blood, CSF, or tissues may suffer from external contamination and thus its levels cannot accurately reflect the internal status of the neurons. Lin et al. showed that the levels of circulating free-form αS and tau do not correlate well with cognitive score based on the minimal mental status exam (MMSE) in PD [212]. A novel platform for biomarker identification is EVs. EVs are lipid bilayer-encapsulated particles secreted by cells. They carry many molecules, including proteins, metabolites, and nucleic acids [239]. CNS-derived EVs can cross the blood–brain barrier and remain stable in the periphery, preventing the CNS biomarkers from being degraded and allowing them to reflect the intraneuronal condition. There are more and more studies on the relationship between EVs and other neurodegenerative dementias. In AD, plasma neural-derived exosomes show significantly higher levels of Aβ1-42 and total tau than controls [240]. A study using neural-derived small EVs showed a specific miRNA signature in AD [241]. In DLB, exosomal αS levels correlate with the severity of cognitive impairment [242]. Exosomal αS levels can distinguish PD from other atypical parkinsonism [243]. The EV-derived neurofilament light chain [244] and phosphorylated insulin receptor 1 [245] are also associated with the severity of rigidity and tremor in PD, respectively. In a recent study, plasma EV αS levels were significantly lower in patients with PD compared with controls, and inversely associated with akinetic-rigidity severity [246]. In another study, the plasma EV αS level was higher in PD patients [247]. The distinction between the two studies may arise from the methods used to purify the heterogeneous population of EVs and the analytical procedures used to detect αS. Other studies suggest that exosomal αS can contribute to interneuronal disease transmission [248,249]. Emerging evidence supports the potential application of EVs as indicators of cognitive impairment; however, the research on the correlation between EVs and PDD is still limited. More studies are required to study the potential diagnostic or prognostic value of EV proteins in PD and PDD.

## 6. Conclusions

Genetic mutations, protein–protein interactions, protein propagation, and an imbalance between synthesis and degradation influence the aggregation propensity of αS. Further clinicopathological correlation and biomarker studies will help to further elucidate the interrelationships of αS pathology in PD and the development of dementia in patients with PD. Moreover, visualizing αS pathology propagation in vivo to assess its temporal relationship to cognitive decline is warranted. These discoveries will be crucial in the development of meaningful disease-modifying or neuroprotective therapies for PDD.
